# P-944. Sniffing Out Antimicrobial Stewardship Opportunities Among Cases of Healthcare onset (HO) Clostridioides difficile infection (CDI)

**DOI:** 10.1093/ofid/ofaf695.1147

**Published:** 2026-01-11

**Authors:** Matthew W Davis, Jeffrey E Topal, Nijad Nashar, Dayna McManus

**Affiliations:** Yale New Haven Hospital, New Haven, Connecticut; Yale New Haven HospitalYale University School of Medicine,, New Haven, Connecticut; Mount Sinai Hospital, New York, NY; Yale New Haven Hospital, New Haven, Connecticut

## Abstract

**Background:**

Healthcare onset (HO) Clostridioides difficile infection (CDI) causes significant increases in morbidity, mortality, and healthcare costs. The Centers for Medicare and Medicaid Services mandates reporting of HO-CDI at all acute care hospitals. Yale New Haven Health System (YNHHS), a 5 hospital system with academic teaching and community medical centers has implemented antimicrobial and diagnostic stewardship to reduce the risk of HO-CDI. To identify additional antimicrobial stewardship (AST) opportunities an audit of HO-CDI cases was performed.

**Methods:**

YNHHS HO-CDI cases from 11/4/2023 to 3/11/2025 were peer reviewed by a team of 1 infectious diseases (ID) attending physician, 2 ID pharmacists and 1 ID pharmacy resident. CDI cases were defined using a 2-step testing algorithm. HO-CDI was defined by National Healthcare Safety Network criteria. See Table 1 for demographic data and risk factors collected.

Following review, CDI cases were classified in one of five categories: CDI with appropriate antibiotic use, CDI with antibiotic use with an opportunity for AST interventions, CDI with antibiotic use with AST interventions which were not accepted, CDI due to an unclear cause without antibiotic exposure within the last 90 days, and CDI which did not meet the above categories.

**Results:**

A total of 100 HO-CDI cases were assessed. 38% of cases were unavoidable: 84% (32/38) were due to appropriate antibiotic use and 16% (6/38) occurred in patients who did not receive antibiotics within the preceding 90 days. 52% of cases were potentially preventable: 71% (37/52) had AST opportunities without intervention, 29% (15/52) of cases had unaccepted AST interventions. Of the potentially preventable cases, 33% (17/52) had an ID consult recommendation for the implicated antibiotic use. The remaining 10% of cases were due to other causes, such as delays in diagnostic testing and outpatient antibiotic use (Table 3).

**Conclusion:**

Although AST interventions can reduce the risk of HO-CDI, we identified that 48% of the HO-CDI cases may not be preventable by common inpatient AST interventions for CDI. Optimizing the impact of stewardship in preventing CDI cases requires collaboration from all care team members, including ID consult services.

**Disclosures:**

All Authors: No reported disclosures

